# A Glycoprotein in Shells of Conspecifics Induces Larval Settlement of the Pacific Oyster *Crassostrea gigas*

**DOI:** 10.1371/journal.pone.0082358

**Published:** 2013-12-12

**Authors:** Hebert Ely Vasquez, Kyotaro Hashimoto, Asami Yoshida, Kenji Hara, Chisato Chris Imai, Hitoshi Kitamura, Cyril Glenn Satuito

**Affiliations:** 1 Graduate School of Fisheries Science and Environmental Studies, Nagasaki University, Nagasaki, Japan; 2 Department of Pediatric Infectious Disease, Institute of Tropical Medicine, Nagasaki University, Nagasaki, Japan; University of New South Wales, Australia

## Abstract

Settlement of larvae of *Crassostrea gigas* on shell chips (SC) prepared from shells of 11 different species of mollusks was investigated. Furthermore, the settlement inducing compound in the shell of *C. gigas* was extracted and subjected to various treatments to characterize the chemical cue. *C. gigas* larvae settled on SC of all species tested except on *Patinopecten yessoensis* and *Atrina pinnata.* In SC of species that induced *C. gigas* larvae to settle, settlement was proportionate to the amount of SC supplied to the larvae. When compared to *C. gigas* SC, all species except *Crassostrea nippona* showed lower settlement inducing activities, suggesting that the cue may be more abundant or in a more available form to the larvae in shells of conspecific and *C. nippona* than in other species. The settlement inducing activity of *C. gigas* SC remained intact after antibiotic treatment. Extraction of *C. gigas* SC with diethyl ether (Et_2_O-ex), ethanol (EtOH-ex), and water (Aq-ex) did not induce larval settlement of *C. gigas* larvae. However, extraction of *C. gigas* SC with 2N of hydrochloric acid (HCl-ex) induced larval settlement that was at the same level as the SC. The settlement inducing compound in the HCl-ex was stable at 100°C but was destroyed or degraded after pepsin, trypsin, PNGase F and trifluoromethanesulfonic acid treatments. This chemical cue eluted between the molecular mass range of 45 and 150 kDa after gel filtration and revealed a major band at 55 kDa on the SDS-PAGE gel after staining with Stains-all. Thus, a 55 kDa glycoprotein component in the organic matrix of *C. gigas* shells is hypothesized to be the chemical basis of larval settlement on conspecifics.

## Introduction

In the life cycle of most marine invertebrates, settlement marks the transition from planktonic larval to sessile adult phase [1]. Planktonic larvae are assumed to be transported like passive particles to given locations as a result of larval dispersal processes controlled by general oceanic circulation [2], [3]. However, more evidence now suggest that larval settlement is a dynamic process where larvae actively respond to a wide range of variables or cues that may provide information to secure a site appropriate for its post-settlement growth and survival [4]. These cues may come from microbial or bacterial biofilms, prey or predator, conspecifics and macroalgal hosts [5], [6].

Many sessile invertebrate species are found primarily in monospecific aggregations. Aggregation clearly facilitates group living by increasing post-metamorphic survival [7]. Proximity also increases fertilization success for both internally fertilizing and freely spawning species [8], [9] and this is particularly true if spawning is synchronized [10]. However, the benefits of living in aggregations also come at a price. In benthic suspension feeders, cannibalism can occur [11], [12]. Aggregated adults must compete for food, which may decrease individual fitness, and reduce individual growth rates [13]. The prevalence of gregariousness in sessile marine communities suggests that benefits outweigh the disadvantages.

Studies have shown that for many species, settlement can be induced by the presence of their conspecifics [1], [14], [15], [16]. Examples of these species include the sipunculan worm *Golfingia misakiana* [17], the polychaete *Hydroides dianthus* [18], the barnacle *Amphibalanus amphitrite* [1], [5], [19], [20], [21], [22], the holothurian *Psolus chitinoides* [23], the northern sand dollar *Echinarachnius parma* [24] and the sea urchin *Tripneustes gratilla* [25]. Of these, *A. amphitrite* has been the most studied species as a model to demonstrate the chemical basis of gregariousness. Matsumura et al. [20] partially characterized the settlement inducing compound from adult *A. amphitrite* as a high molecular weight protein complex with four subunits and coined the term SIPC or Settlement Inducing Protein Complex. More recently, Dreanno et al. [26] reported that SIPC of *A. amphitrite* is a glycoprotein that shares a 30% sequence homology with the thioester containing family of proteins that includes the alpha (2)-macroglobulins. Studies are now focused on elucidating the molecular structure of the SIPC and their receptors, with the future goal of understanding the evolution of barnacles and their settlement behavior.

Oysters are also known to form monospecific aggregations that are commonly called oyster reefs. Gregarious behavior in oysters was first reported by Cole and Knight-Jones [27] for *Ostrea edulis*. Bayne [28] later confirmed observations by Cole and Knight-Jones [27] and reported that extracts of *O. edulis* tissue were effective in promoting larval settlement when applied to a surface. Since then, the inducing effect of conspecifics on larval settlement has been studied for other oyster species i.e., *Crassostrea virginica* [29]–[33], *C. gigas* [34], [35], and *C. ariakensis* [36], although the bulk of information is reported on *C. virginica*. In *C. virginica*, adult shells, water preconditioned by adults and “oyster shell liquor’ have been shown to promote larval settlement [29], [32], [33]. Veitch and Hidu [31] further reported that the inducing substance present in the “oyster shell liquor” was a thyroxine containing protein with MW greater than 100,000 Da. Crisp [29] observed that larvae of *C. virginica* settled almost entirely on conspecific shells but destruction of the organic layers on its surface clearly rendered the substratum unfavorable. Field experiments conducted by Diederich [37] also confirmed that recruitment of *C. gigas* larvae was higher on conspecifics and that recruitment was the same on both living substrate and dead oyster shells. In hatcheries in California (USA), France and Latin America, setting oyster larvae on shell chips of conspecifics is the commonly used method of producing cultchless spats [38]–[41], a method based on the gregarious settlement behavior of oyster larvae. It is apparent that chemical cues from conspecifics and the involvement of these cues in the gregarious behavior of *C. gigas* larvae during settlement are still poorly understood, and the elucidation of these cues will lead to the clarification of the larval settlement mechanism of *C. gigas*. Furthermore, newly identified larval settlement inducer compounds can also find application in the development of *C. gigas* aquaculture.

In the present study, we investigated the settlement inducing activities of shell chips (SC) prepared from 11 different molluscan species on larvae of *C. gigas*. The purpose was to check if *C. gigas* larvae exhibit substrate specificity during settlement. Furthermore, the authors attempted to extract and partially characterize the settlement inducing compound in shells of adult *C. gigas.*

## Materials and Methods

### Broodstock and Larval Culture

Adult *Crassostrea gigas* used for spawning were purchased from Konagai Fisheries Cooperative (Nagasaki, Japan). These oyster were from an oyster culture farm in Konagai-cho, Nagasaki, Japan (32°55′08′′N, 130°11′42′′E). These were maintained in an aquarium inside the laboratory and were fed daily with a combination of *Chaetoceros gracilis* and an artificial feed for bivalves (M1, Nosan Corp., Yokohama, Japan). During the spawning season, adults were kept at 20±1°C. This was to suppress natural spawning of the broodstock. During the winter season, adults were maintained at 25±1°C to allow gonad development and maturation. Thirty different broodstock groups were used for spawning to obtain larvae for the settlement assays conducted during the period between 2008 and 2013.

Gonads were stripped from the oysters to collect gametes [42]. Eggs and sperm were separately suspended in 2L glass beakers containing GF/C (Whatman glass fiber filter; pore size: 1.2 µm) filtered sea water (FSW) adjusted to 24°C. Eggs were washed several times with FSW through repeated decantation and were then fertilized with a small volume of the sperm suspension. Thirty minutes after artificial fertilization, fertilized eggs were collected in a 20 µm net, washed four to five times with FSW and re-suspended in 2 L glass beakers containing FSW. Fertilized eggs were kept at 24±1°C in an incubator for 24 h.

After 24 h, swimming straight hinged larvae were collected in a 40**µm net, gently washed with FSW, stocked in 2 L glass beakers at an initial density of 5 larvae mL^−1^ and cultured in a water bath at 24±1°C. Larvae were fed daily with the following algal diet: *Chaetoceros calcitrans* (5×10^4^ cells mL^−1^) from the 1^st^ to the 5^th^ day of culture, *C. calcitrans* (2.5×10^4^ cells mL^−1^) and *C. gracilis* (2.5×10^4^ cells mL^−1^) from the 6^th^ to the 10^th^ day of culture, and *C. gracilis* (5×10^4^ cells mL^−1^) from the 11^th^ day onward during the culture period. Cultures were aerated (20 mL min^−1^) and the water renewed daily throughout the culture period. Cultures were kept in a dark environment but were exposed to light daily for approximately 1 h during water changing. Salinity of seawater used was 32 psu. Larvae usually reached the pediveliger stage on the 17 to 18 days after fertilization. Pediveligers employed in assays were between 24 and 28 days old after fertilization and ranged from 300 to 320 µm in shell length.

### Larval Settlement Assays

Twenty pediveliger larvae were released into each petri dish (ø 60 mm×15 mm height) containing 20 mL FSW and the substrate (shell chips (SC), GF/C filter paper (ø 47 mm, GF/C) or GF/C containing the test compound). Settlement inducing activities of the different substrates were evaluated by the number of individuals that metamorphosed to post larvae within 24 h. Post larvae were confirmed under the microscope as individuals that secreted cement substance or those with post-larval shell growth. Petri dishes, each containing 20 pediveligers and 20 mL of FSW only or with GF/C as a substrate were set as the blank control. All assays were conducted in a dark environment at 24±1°C in an incubator.

**Figure 1:**
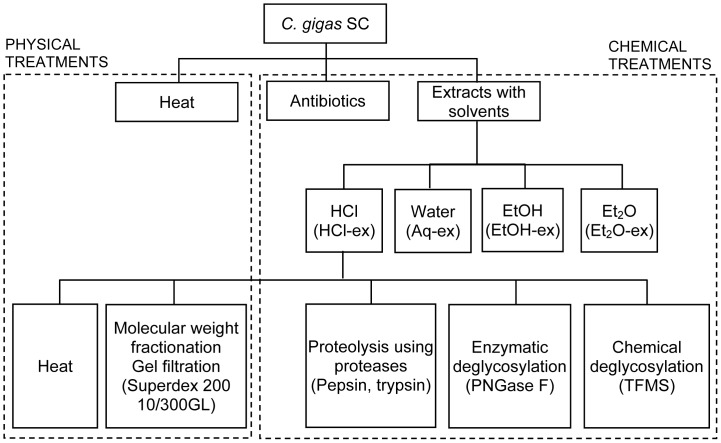
Experimental design for the characterization of the settlement inducer in *C. gigas* SC.

In assays with SC of the 11 species of mollusks, tests were conducted at 0, 10, 50 and 100 mg dry weight of SC for each species. SC of *C. gigas* were further subjected to several bioassay-guided treatments using physical and chemical methods as indicated in the flowchart in Fig. 1.

In the assay on heat treatment of *C. gigas* SC, the amounts tested were 50 mg and 500 mg per petri dish. In the assay on antibiotic treatment of *C. gigas* SC, the amount tested was 50 mg per petri dish.

In assays to investigate the activities of *C. gigas* SC extracts (aqueous, HCl, ethanol and diethyl ether) and the effects of chemical (proteolysis using proteases and deglycosylation using PNGase F and TFMS) and physical (heat and molecular weight fractionation) treatments on the activity of *C. gigas* HCl extract (HCl-ex), amounts assayed were expressed in weight equivalent of SC, where 1 mg SC was equivalent to 0.5 µg of protein as determined after measuring using the Lowry assay [43].

The extracts or treated extracts were applied to GF/C (ø 47 mm), dried and taped to the bottom of each petri dish using double-faced adhesive tape. For each type of SC, extract, fraction and treatment, at least six replicate experiments were conducted using larvae from at least two separate culture batches. In all assays with *C. gigas* SC extracts, 50 mg of *C. gigas* SC per petri dish was set as a positive control.

### Preparation of Shell Chips (SC) of the 11 Molluscan Species and Treated (Heat and Antibiotics) *C. gigas* SC

SC of the 11 species of mollusks were prepared using shells taken from living specimens. The taxonomic classifications and origins of specimens used to obtain the shells are described in Table 1. Prior to the preparation of SC of each species, shells were scrubbed using a metal wire brush, to remove attached organisms and visible traces of muscle tissues, washed and then dried. Dried shells were crushed using a mallet and crushed shell fragments were sieved through two metallic mesh screens; first, 1.0 mm mesh size screen, and then the 0.5 mm screen. Shells fragments that remained on the 0.5 mm mesh screen were collected and used as SC. SC were stored in a freezer at −40°C until these were used in experiments.

**Table 1:** Taxonomy of the 11 species of mollusks used as specimens.

Class	Order	Family	Species	Specimen origin
Bivalvia	Ostreoida	Ostreidae	*Crassostrea gigas*	Konagai fishing port, Nagasaki, Japan
		Ostreidae	*C. nippona*	Nagasaki Prefectural Institute of Fisheries, Nagasaki, Japan
		Ostreidae	*C. iredalei*	Manila Bay, Philippines
		Ostreidae	*Ostrea circumpicta*	Hiroshima Prefectural Technology Research Institute, Hiroshima, Japan
		Ostreidae	*O. denselamellosa*	Oita Prefectural Agriculture, Forestry and Fisheries Research Center, Oita, Japan
		Ostreidae	*Saccostrea kegaki*	Koebaru, Nagasaki, Japan
		Ostreidae	*S. mordax*	Koebaru, Nagasaki, Japan
	Pectinoida	Pectinidae	*Patinopecten yessoensis*	Okasei Fishing Company, Fukuoka, Japan
	Pterioida	Pteriidae	*Pinctada fucata martensii*	Nagasaki Prefectural Institute of Fisheries, Nagasaki, Japan
		Pinnidae	*Atrina pinnata*	Nagasaki Prefectural Institute of Fisheries, Nagasaki, Japan
Gastropoda		Haliotidae	*Haliotis discus*	Nagasaki Prefectural Institute of Fisheries, Nagasaki, Japan

In the heat treatment experiment, *C. gigas* SC heated at 100°C and 200°C for 1 h and at 300°C for 3 h in an electrical oven were prepared and used in assays.

In the antibiotic treatment experiment, *C. gigas* SC were soaked in 1AB and 10AB concentrations of antibiotic solutions for 48 h following the method used by Yang et al. [44] for sterilization of macroalgae. Antibiotic treated SC were rinsed six times using a total volume of 2 L FSW prior to use in assays. Concentrations of the drugs in 1AB of antibiotic solution were: 20 mg L^−1^ of streptomycin sulphate, 10 mg L^−1^ of penicillin G, 2 mg L^−1^ of neomycin and 10 mg L^−1^ of kanamycin, and concentrations were increased ten-fold in the 10AB solution.

### Preparation of *C. gigas* SC Extracts

#### Aqueous extract (Aq-ex)

Ten grams of *C. gigas* SC was placed in a mortar and ground while adding small amounts of distilled water (DW) warmed to 60°C into the mortar. The supernatant was collected and the procedure repeated until a total of 100 mL of the supernatant was collected. The supernatant was filtered through GF/C and then used in assays. After extraction with DW, residues were collected on a glass petri dish, completely dried in an oven at 50°C and then used in assays.

#### Ethanol extract (EtOH-ex)

Ten grams of *C. gigas* SC was soaked overnight in 100 mL of ethanol. The supernatant was then collected, filtered through GF/C and concentrated to 1 g mL^−1^ SC equivalent using a rotary evaporator. Concentrated EtOH-ex was then applied to GF/C, air-dried and used in assays. After extraction with ethanol, residues were also collected on a glass petri dish, air-dried at room temperature for approximately 2 h until the solvent was completely evaporated. Dried residue was then used in assays.

#### Diethyl ether extract (Et_2_O-ex)

Diethyl ether extract was prepared in the same manner as EtOH-ex. After extraction with diethyl ether, residues were also collected on a glass petri dish and air-dried at room temperature. Dried residue was then used in assays.

#### Hydrochloric acid extract (HCl-ex)

A total amount of 150 g of *C. gigas* SC was completely dissolved in 1L of 2 N HCl [45]. This solution was centrifuged at 12859×g for 20 min at 4°C, the supernatant was collected and filtered through GF/C and then dialyzed against 0.01N HCl solution at 4°C for a period of three days until the final pH of the HCl-ex was 6. The HCl-ex was stored in the freezer until used in assays. Freeze dried samples of HCl-ex (FD HCl-ex) were also prepared and stored as powder in the freezer until used in assays. Activities of extracts were examined as described in the larval settlement assays. Protein contents of both HCl-ex and FD HCl-ex were determined by the Lowry assay [43].

Ethanol, diethyl ether and HCl were purchased from Wako Pure Chemical Co. (Osaka, Japan).

### Treatments of FD HCl-ex

Freeze-dried HCl-ex (FD HCl-ex) samples were used in all treatment assays. FD HCl-ex was dissolved in distilled water to desired concentration prior to use.

In the heat treatment experiment, FD HCl-ex solutions were heated at 60°C and 100°C for 30 min in a water bath, cooled and applied to the GF/C. The GF/C were then dried and taped to the bottom of petri dishes.

Pepsin (Sigma, St. Louis MO, USA) and trypsin (Wako Pure Chemical Co., Osaka, Japan) treatments of FD HCl-ex were done following the method of Jouuchi et al. [46] with slight modifications. Pepsin was dissolved at a concentration of 1 mg mL^−1^ in diluted HCl (pH 2). A counterpart solution without pepsin was also prepared. GF/C containing FD HCl-ex (100 mg SC equivalent) were soaked in 30 mL solutions with and without 1 mg mL^−1^ pepsin for 4 h at 37°C. Trypsin was dissolved at concentrations of 1 and 10 mg mL^−1^ in phosphate buffer (pH 7.6). A counterpart solution without trypsin was also prepared. GF/C containing FD HCl-ex (100 mg SC equivalent) were soaked in 30 mL solutions with and without the trypsin for 2 h at 37°C. After the pepsin and trypsin treatments, GF/C were washed twice, first in 1 L DW and then in 1 L FSW, dried and assayed.

FD HCl-ex was also subjected to enzymatic deglycosylation with peptide-N-glycosidase F (PNGase F, Sigma-Aldrich, St. Louis MO, USA). Twenty-five µL of phosphate buffer (100 mM, pH 7.5) was added to an equivalent volume of 200 µg of FD HCl-ex solution. The mixture was then incubated with 10 IU of PNGase F at 37°C for 24 h [46]. A counterpart solution was also prepared but without PNGase F. After 24 h, FD HCl-ex solutions with and without the PNGase F were applied to GF/C, and the GF/C washed as described above, dried and then assayed. FD HCl-ex was also subjected to chemical deglycosylation using trifluoromethanesulfonic acid (TFMS, Wako Pure Chemicals Co., Osaka, Japan) following the manufacturer’s instruction manual. Equal amounts of FD HCl-ex solution (0.5 mL) and TFMS (0.5 mL) were mixed and let to react for 1 h on ice. After 1 h, 0.5 mL of 1 M Tris was added to the solution, and was dialyzed overnight against DW to remove TFMS from the mixture. A counterpart solution was also prepared in the same manner but without TFMS. After dialysis, FD HCl-ex treated with TFMS and its counterpart solution were applied to GF/C, washed as described above, dried and then assayed.

### Gel Filtration of FD HCl-ex

FD HCl-ex powder was dissolved in milliQ filtered sterile water (MilliQ Millipore Water System, Bedford MA, USA), applied to a Superdex 200 10/300 GL column equilibrated with 0.5 M NaCl using a 500 µL loop, and eluted with the same buffer at a rate of 0.5 mL min^−1^, using the HPLC system. A total of 35 tubes of 0.5 mL fractions were collected, pooled into 3 molecular size range fractions and then assayed. The pooled fractions were also subjected to SDS-PAGE.

### SDS PAGE of FD HCl-ex

Protein contents of each of the FD HCl-ex and fractions were initially measured by UV absorption at 280 nm [47]. Next, 10 µg protein content of FD HCl-ex and its pooled fractions were each dissolved in 30 µL distilled water and then homogenized in 30 µL of sample buffer containing 125 mM tris(hydroxymethyl)aminomethane HCl (pH 6.8), 4% sodium dodecylsulfate (SDS), 10% 2-mercaptoethanol, 0.004% bromophenol blue and 10% glycerol. The homogenates were then boiled for 10 min. After centrifugation at 1744×g for 10 min, FD HCl-ex and pooled fractions homogenates were used directly for analysis by polyacrylamide gel electrophoresis containing SDS (SDS-PAGE) according to Laemmli’s [48] method. Aliquots (20 µL) of samples were electrophoresed on a separation gel containing 10% acrylamide with a stacking gel of 3% acrylamide at 20 mA for 1.5 h. Protein profiles of samples were visualized with Stains-all (Sigma-Aldrich, St. Louis MO, USA).

### Statistical Analysis

Settlement inducing activities of the different amounts and species of SC, extracts (before and after treatments) and fractions were evaluated by the number of post larvae that settled and percentages were presented as arithmetic means with standard deviations (SD). Data were analyzed using binomial generalized linear models (GLM) or quasi-binomial GLM when a model resulted in overdispersion. Post hoc Tukey HSD multiple comparison test was conducted to assess differences in settlement inducing activities between samples in the model. Wald test [49] was used for pairwise comparisons. In the analysis for settlement inducing activities of SC of the different molluscan species at 10, 50 and 100 mg the model included species and weight of SC as variables, and analysis of variance (ANOVA) was performed to check for interaction effects between these two factors. Estimated settlement differences by percentage for SC of each of the 10 species of mollusks compared to *C. gigas* SC were calculated based on odds ratios derived from the model results. All statistical analysis were carried out using the statistical package R (R Foundation for Statistical Computing: http://www.r-project.org, ver. 3.0.1) [50]. Differences were considered significant at p<0.05.

## Results

The percentage of post larvae of *C. gigas* on clean petri dishes with FSW (blank control) was 1.1±4.3%.

### Settlement Inducing Activities of SC of the 11 Species of Mollusk

**Figure 2:**
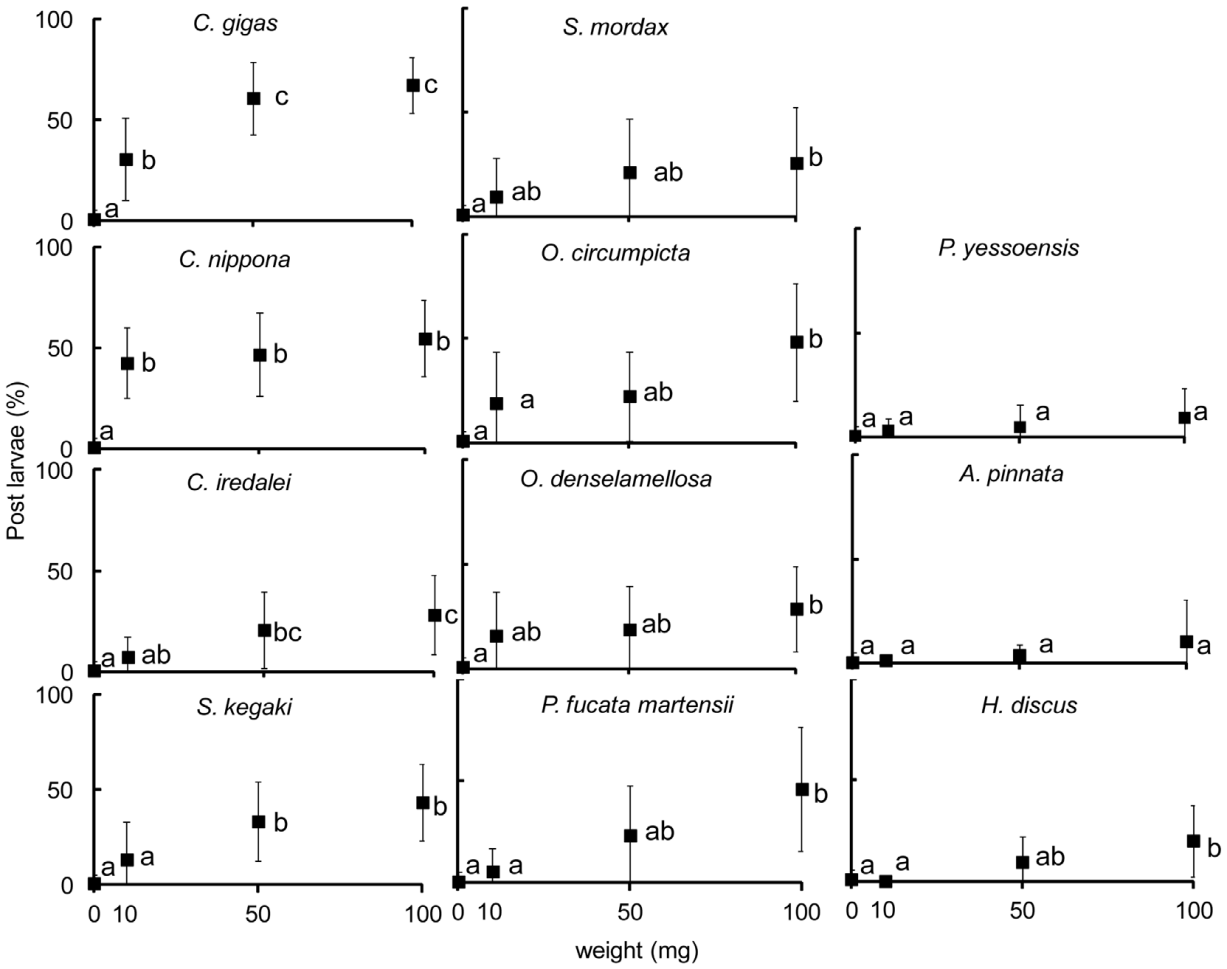
Percentages of post larvae that settled on SC of the different species of mollusks. Closed squares are means of 6 to 30 replicates and error bars represent standard deviation (SD). Letters indicate results of the post hoc Tukey HSD test of activities within species at 0, 10, 50 and 100 mg. Values connected by the same letter are not significantly different (p≥0.05).

Percentages of *C. gigas* post larvae at 0, 10, 50 and 100 mg SC prepared from 11 different species of mollusks are as shown in Fig. 2. Settlement inducing activities of the different SC were assessed with Tukey HSD test for multiple comparisons after all data were fitted in the quasi-binomial model. Of the 11 different species, SC of *P. yessoensis* and *A. pinnata* did not induce settlement of *C. gigas* larvae at all weights tested. On the other hand, SC of *S. mordax*, *O. circumpicta*, *O. denselamellosa*, *P. fucata martensii* and *H. discus* induced larval settlement only at 100 mg, while *C. iredalei* and *S. kegaki* induced settlement at 50 and 100 mg of SC. Percentages of post larvae on SC of *C. gigas* and *C. nippona* were significantly higher than those of the blank control at all weights tested (Fig. 2). For species that induced larval settlement of *C. gigas*, percentages of post larvae increased with the weight of SC used and were highest at 100 mg. Analysis of variance also showed that the settlement inducing activity of SC was significantly affected by both species (<0.0001) and weight (<0.0001) (Table 2). However, the interaction between species and weight of SC did not significantly improve the model (p = 0.2566) (Table 2) and hence, not included as a variable in the final GLM model.

**Table 2:** ANOVA result of the effect of species and amount of SC on *C. gigas* larval settlement.

	Df	Deviance	ResidualDf	ResidualDeviance	p
null (intercept)			383	3149.4	
Species	10	1041.65	373	2107.8	<0.0001
Amount	1	383.33	372	1724.4	<0.0001
Species×Amount	10	53.13	362	1671.3	0.2566

Statistics of the quasi-binomial GLM applied to the output for dependent variable larval settlement. Species refers to the 11 species of SC tested; amount refers to the weight of SC.

**Figure 3:**
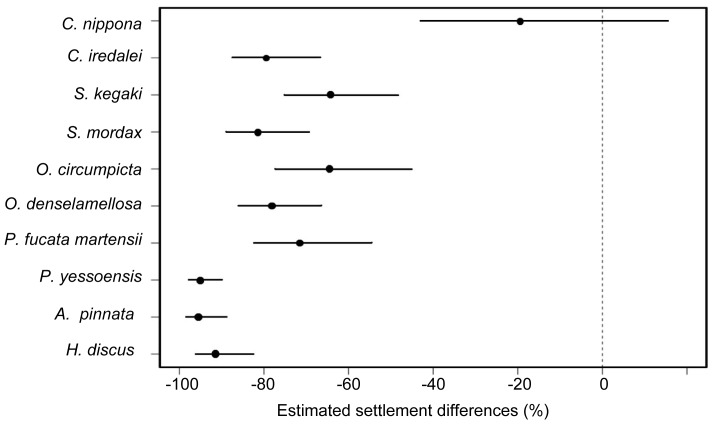
Estimated percent settlement differences of the 10 molluscan species SC *vs. C. gigas* SC (0). Closed circles represent mean estimates and error bars are 95% confidence intervals. Estimated settlement differences between the ten molluscan species and *C. gigas* (as the reference) were calculated based on the odds ratios.

Estimated settlement differences (%) of each of the 10 species of mollusks compared to *C. gigas* were calculated from the final GLM model and plotted in Fig. 3. Of the 10 species of SC, only *C. nippona* showed a settlement inducing activity that was not significantly different from that of *C. gigas*. By contrast, SC of the other nine species all showed lower settlement inducing activities, and estimated settlements on SC of these species were 60 to 95% less than that of *C. gigas*. Except for *P. fucata martensii*, estimated settlements of *C. gigas* larvae on SC of species not belonging to Osteridae were 90% less than that of conspecific SC (Fig. 3).

### Effect of Heat and Antibiotics Treatments on the Activity of *C. gigas* SC

**Figure 4:**
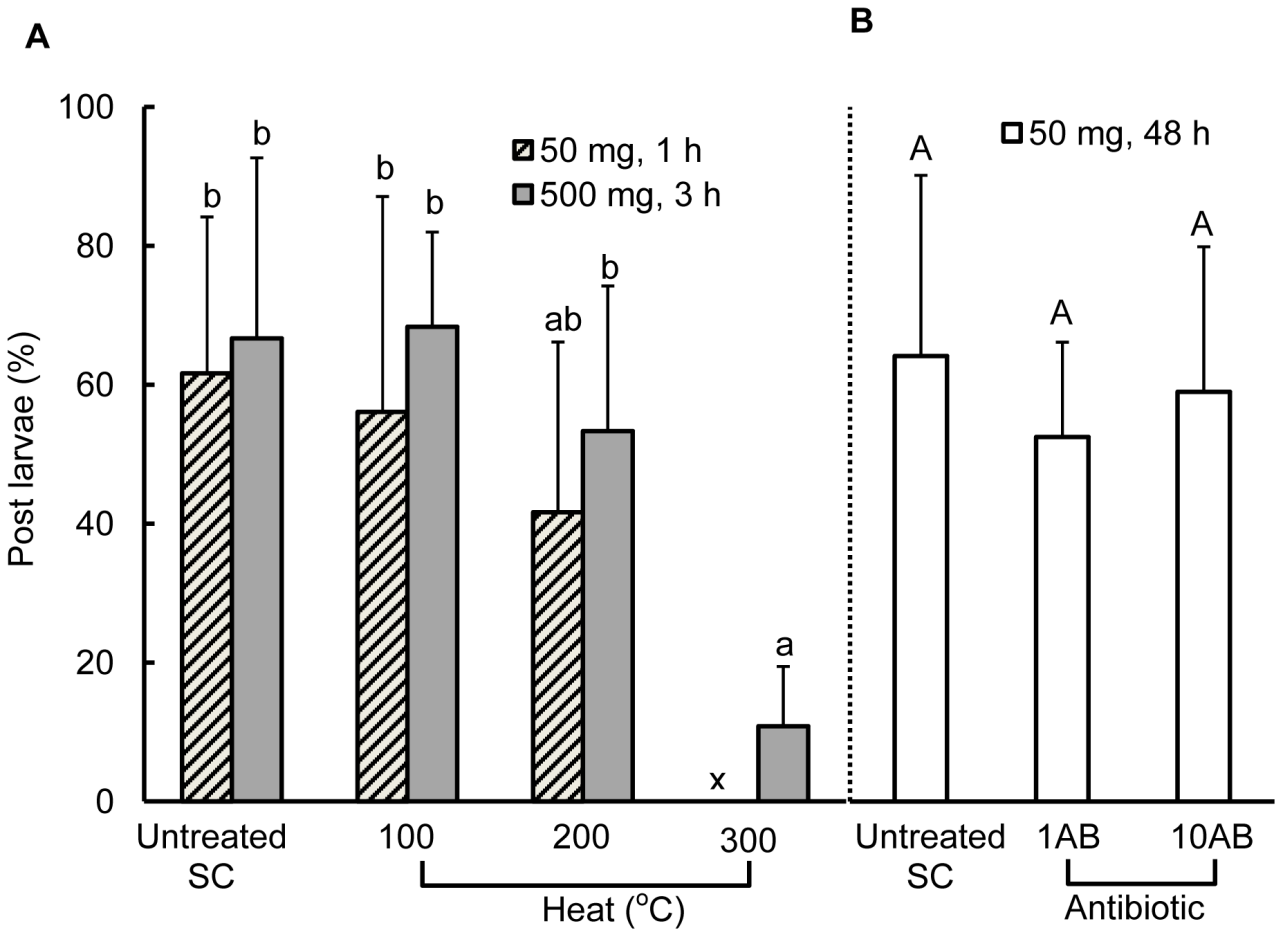
Percentages of post larvae on *C. gigas* SC that were heated at different temperatures (A), and treated with antibiotic solutions (B). A: Boxes with slanting lines and shading indicate results of heat treatment experiments of 50 mg SC and 500 mg SC, respectively. x indicates no data. B: blank boxes indicate the antibiotic treatment experiment of 50 mg SC. 1AB = streptomycin sulphate 20 mg L^−1^, penicillin G 10 mg L^−1^, neomycin 2 mg L^−1^, and kanamycin 10 mg L^−1^, and 10AB = 1AB concentration increased tenfold. Data are means of 6 to 9 replicates and error bars represent standard deviations (SD). Lowercase and uppercase letters indicate results of the post hoc Tukey HSD test on heat and antibiotic treatments, respectively. Groups connected by the same letter are not significantly different (p≥0.05).

The effect of heat treatments for 1 h and 3 h on the activity of *C. gigas* SC is shown in Fig. 4-A. Tukey HSD test showed that up to 200°C, heat had no effect on the settlement inducing activity of *C. gigas* SC, which remained constant regardless of the heating time (1 h and 3 h) and the amount of SC (50 mg and 500 mg) heated. However, the activity of 500 mg SC significantly decreased after heating at 300°C for 3 h. No difference in activity was also found between 50 mg SC heated at 200°C for 1 h and 500 mg SC heated at 300°C for 3 h. The effect of antibiotics treatment on the activity of *C. gigas* SC is shown in Fig. 4-B. Tukey HSD test also showed that treatment with 1AB and 10AB concentrations of antibiotics solution for 48 h did not affect the activity of *C. gigas* SC.

### Settlement Inducing Activities of *C. gigas* SC Extracts

**Figure 5:**
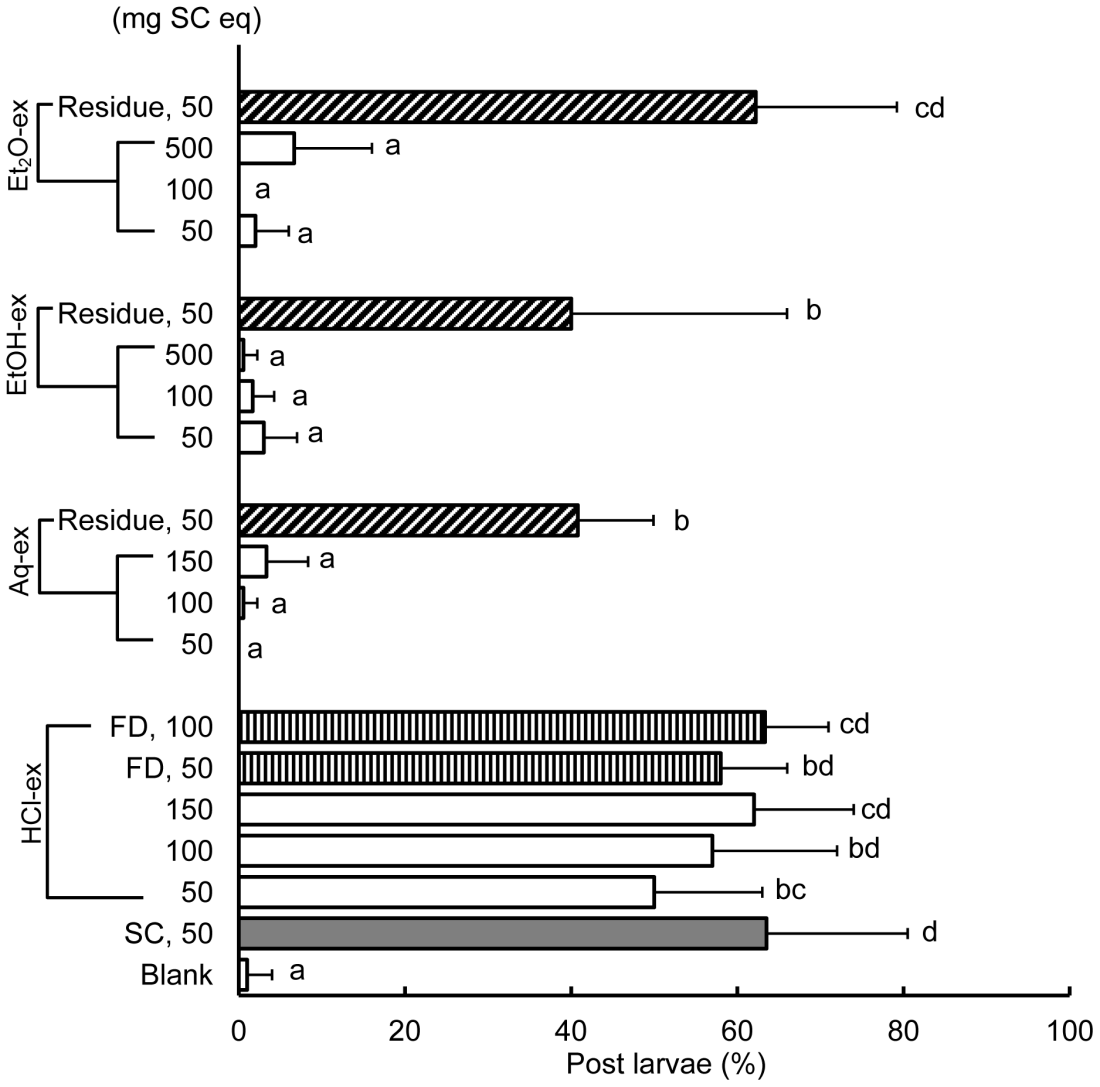
Percentages of post larvae on Et_2_O-ex, EtOH-ex, Aq-ex, HCl-ex and residues after extraction of *C. gigas* SC. Amounts assayed are expressed in mg equivalent of SC (mg SC eq), where each weight equivalent of the extract corresponds to the amount of SC extracted. Et_2_O-ex, EtOH-ex, Aq-ex and HCl-ex indicate *C. gigas* SC extracts with diethyl ether, ethanol, water and hydrochloric acid, respectively. FD HCl-ex indicates freeze dried HCl-ex. Data are means of 6 to 15 replicates and error bars represent standard deviations (SD). Letters indicate results of the post hoc Tukey HSD test. Groups connected by the same letter are not significantly different (p≥0.05).

The settlement inducing activities of Et_2_O-ex, EtOH-ex, Aq-ex, HCl-ex and FD HCl-ex are shown in Fig. 5. Percentages of post larvae in Et_2_O-ex, EtOH-ex and Aq-ex at all concentrations tested were <8% at the highest, and were not significantly different from the blank control as assessed in the Tukey HSD test. The percentage of post larvae on the SC residue of diethyl ether extraction was 62% and was at the same level as the positive control (50 mg SC). On the other hand, settlement inducing activities of SC residues of ethanol and water extractions decreased as compared to the positive control, and their percentages of post larvae were 40% and 41%, respectively, indicating that the extraction process significantly changed the activity of SC.

HCl-ex and FD HCl-ex showed high settlement inducing activities at all concentrations tested; percentages of post larvae were all significantly higher than the blank control and were at the same level as the positive control (50 mg SC), except for the 50 mg SC eq of HCl-ex. Freeze drying did not also affect the activity of HCl-ex; no difference was observed in the percentages of post larvae between HCl-ex and FD HCl-ex at any concentration (Fig. 5).

### Effect of Treatments on the FD HCl-ex Activity

Percentage of post larvae of FD HCl-ex (100 mg SC eq) prior to heating was 64%, while those after heating at 60°C and 100°C for 30 min were 56% and 62%, respectively. Tukey HSD test revealed no difference in the settlement inducing activities between the three groups (p≥0.05).

**Figure 6:**
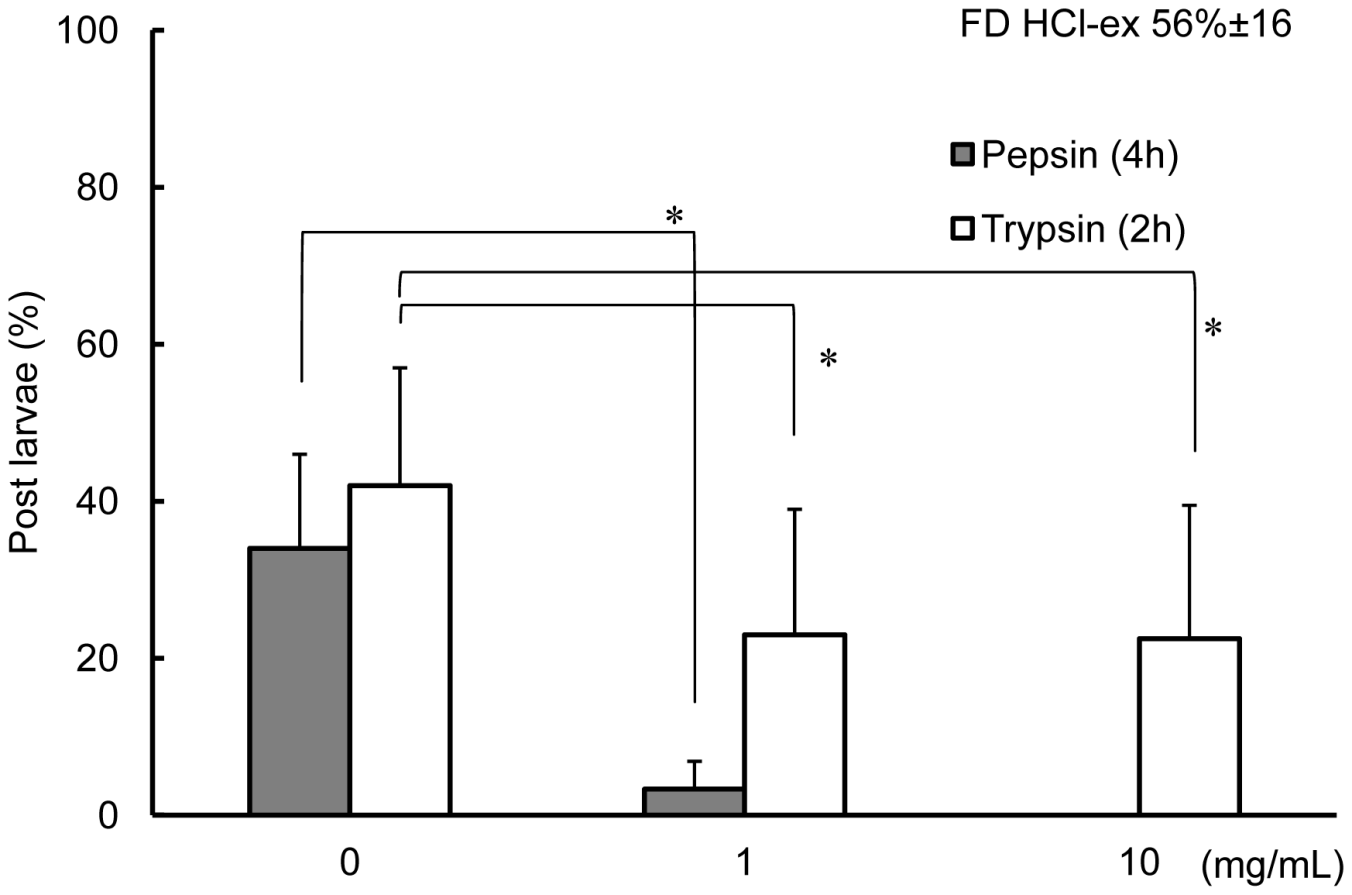
Percentages of post larvae on pepsin and trypsin treated FD HCl-ex of *C. gigas* SC. Shaded and open boxes represent pepsin and trypsin treated experiments, respectively. Data are means of 9 replicates and error bars represent standard deviations (SD). Lines connected groups that were compared using Wald test. Asterisks * indicate significantly different groups (p<0.05).

The effects of pepsin and trypsin treatments on the activity of FD HCl-ex are as shown in Fig. 6. Activity of FD HCl-ex significantly decreased after treatment with 1 mg mL^−1^ of pepsin (p<0.05, Wald test); percentages of post larvae of FD HCL-ex before and after treatment with 1 mg mL^−1^ pepsin were 35% and 3%, respectively. Activities of FD HCl-ex treated with 1 and 10 mg mL^−1^ of trypsin also significantly decreased as compared to the control (0 mg mL^−1^ trypsin) (p<0.05, Wald test), which was FD HCl-ex subjected to the same treatment procedure but without trypsin.

**Figure 7:**
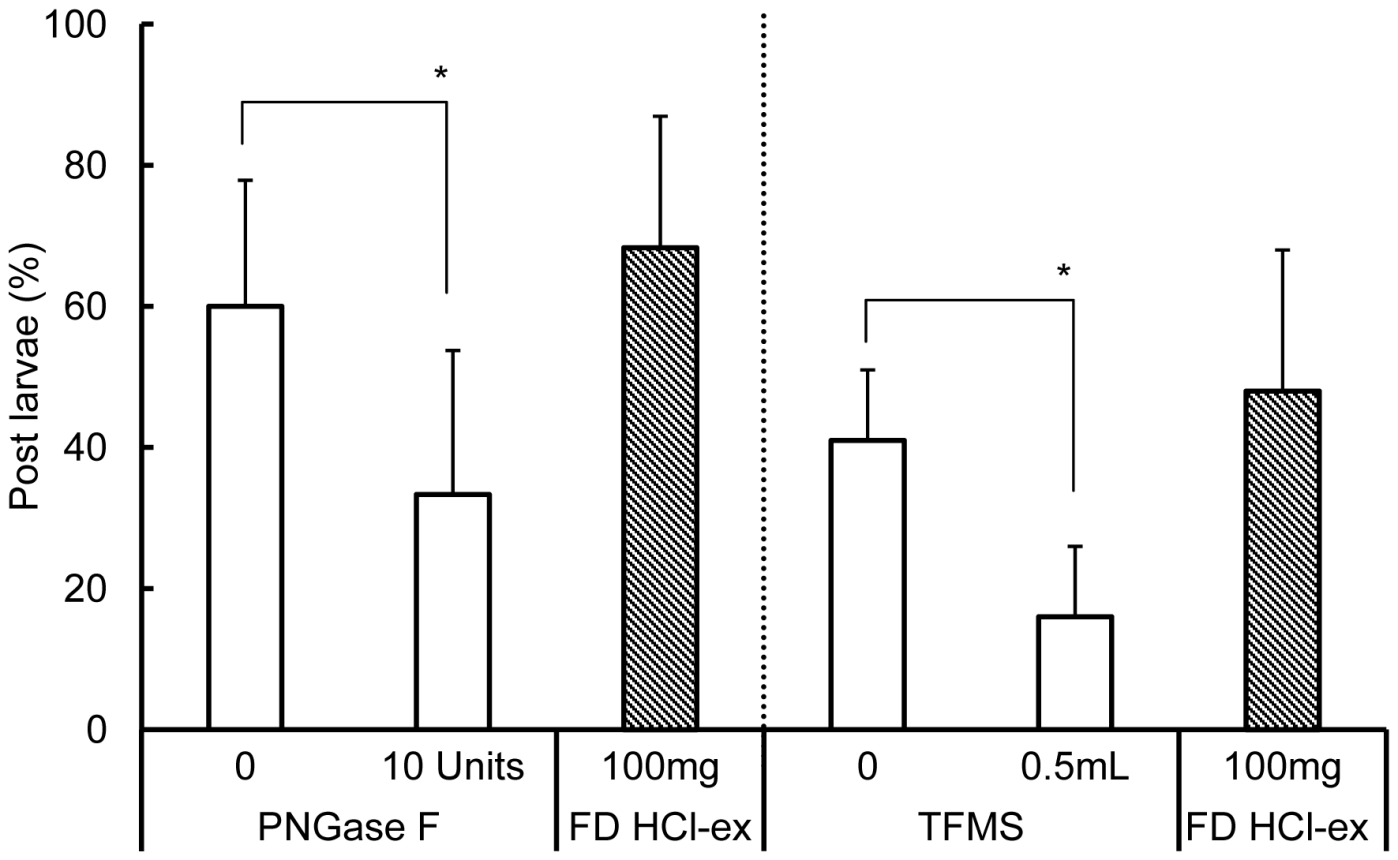
Percentages of post larvae on PNGase F and TFMS treated FD HCl-ex of *C. gigas* SC. Boxes with slanting lines indicate post larvae (%) settled on FD HCl-ex 100 mg SC eq subjected to the same treatment procedure but without trypsin. Data are means of 9 replicates and error bars represent standard deviations (SD). Lines connected groups that were compared using Wald test. Asterisks * indicate significantly different groups (p<0.05).

The effects of deglycosylation using PNGase F and TFMS on the activity of FD HCl-ex are as shown in Fig. 7. The activity of FD HCl-ex after treatment with 10 IU of PNGase F significantly decreased as compared to the control (0 IU PNGase F) (p<0.05, Wald test), which was FD HCl-ex subjected to the same treatment procedure but without PNGase F. The activity of FD HCl-ex after treatment with 0.5 mL of TFMS also significantly decreased as compared to the control (0 mL TFMS) (p<0.05, Wald test), which was FD HCl-ex subjected to the same treatment procedure but without TFMS.

### Activities of Fractions of FD HCl-ex from Gel Filtration

**Figure 8:**
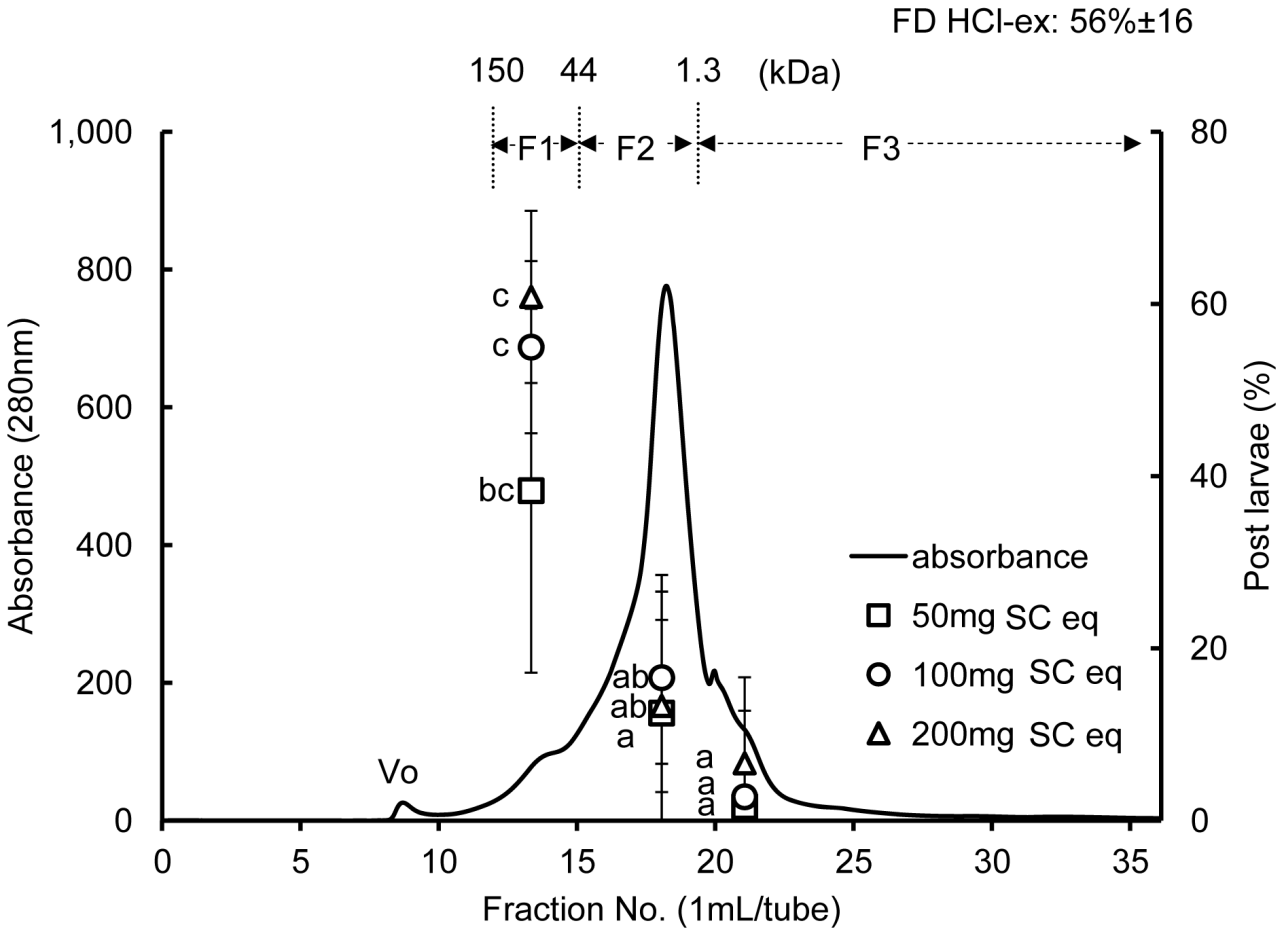
Absorbance and eluted fractions of FD HCl-ex of *C. gigas* SC after filtration in Superdex 200 10/300 GL column, and the percentages of post larvae at 50, 100 and 200 mg SC equivalent of each of the pooled fractions (F1: 45–150 kDa, F2:1.3–44 kDa, and F3:<1.3 kDa molecular mass ranges). Vo indicates void volume. Data are means of 6 to 9 replicates and error bars represent standard deviations (SD). Letters indicate results of the post hoc Tukey HSD test. Values connected by the same letter are not significantly different (p≥0.05).

The distribution of FD HCl-ex following gel filtration and percentages of post larvae of the pooled fractions are as shown in Fig. 8.

Eluted fractions after filtration of FD HCl-ex in Superdex 200 10/300 GL column exhibited one peak with several shoulders. Eluted fractions were pooled into three, each with corresponding MW range of: 45 to 150 kDa for fraction 1; 1.3 to 44 kDa with an eluted peak at approximately 6 kDa for fraction 2; and <1.3 kDa for fraction 3 (Fig. 8).

**Figure 9:**
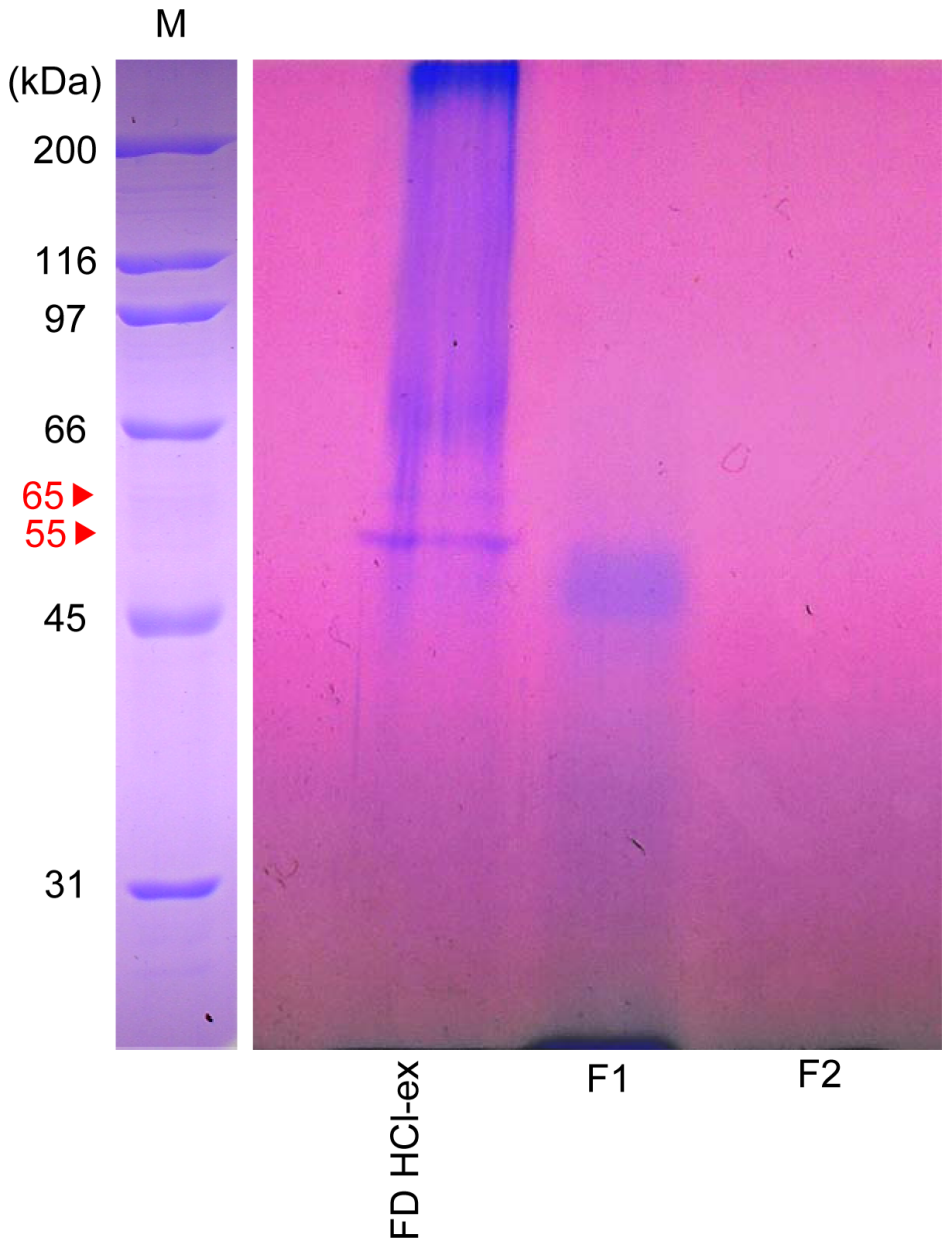
SDS-PAGE gel image of *C. gigas* FD HCl-ex, F1 (fraction 1) and F2 (fraction 2) stained with Stains-all. SDS-PAGE was performed on 10% polyacrylamide gels; 10 µg protein content was loaded in each lane. M indicates the molecular weight markers. Molecular weights were estimated using the molecular weight marker “Broad Range” (BIORAD).

Tukey HSD test showed that percentages of post larvae in response to the different concentrations of fraction 1 (F1) were all significantly higher than that of the blank control but no difference in settlement inducing activity was found between the concentrations tested. Tukey HSD test also revealed that larval settlement on all concentrations of fraction 2 (F2) and fraction 3 (F3) were the same as that of the blank control (FSW). On the other hand, no difference in the settlement inducing activities was found between 50 mg SC eq of F1 and 100 and 200 mg SC eq of F2. The percentage of post larvae in F1 at 100 mg SC eq was 55%, and this was at the same level as the activity of 100 mg SC eq of FD HCl-ex (56%) before gel filtration (p≥0.05, Tukey HSD test) (Fig. 9).

### SDS-PAGE of FD HCl-ex and Fractions from Gel Filtration

The SDS-PAGE gel reveals the protein bands of *C. gigas* FD HCl-ex and fractions 1 and 2 (Fig. 9). After staining with Stains-all (Sigma-Aldrich, St. Louis MO, USA), two bands were visualized in FD HCl-ex lane; a minor band with MW of 65 kDa and a major band with 55 kDa MW. In fraction 1 lane, only one major band with MW of 55 kDa was visualized. No band was visualized in the lane of fraction 2 since the peak eluted in this fraction had a low molecular weight (approximately MW 6 kDa).

## Discussion and Conclusion

In the present investigation, we demonstrated that larvae of the Pacific oyster *C. gigas* differentially settle on shells of different species of mollusks (Fig. 2), albeit in higher numbers on conspecific shells and those of *C. nippona* (Fig. 3). This finding suggests that larvae may recognize shells of their own or related species during settlement and will respond favorably by settling in high numbers. This is consistent with field observations of Diederich [37] where recruitment of *C. gigas* larvae was higher on shells of conspecifics than those of *Mytilus edulis*. Diederich [37] interpreted this observation as the difference in the texture of shells and that larvae exhibited preference for rough oyster shells over smooth shells of mussels. Crisp [29], however, suggested that chemical cues in shells maybe involved since *C. virginica* larvae settled almost entirely on conspecific shells but the destruction of the organic layers on the surface of shells clearly made the substratum unfavorable. In species that induced *C. gigas* larvae to settle, larval settlement was proportionate to the amount of SC given to the larvae (Fig. 2). Thus, increasing the amount of SC also increases the likelihood of larvae to detect the settlement cue, resulting in a higher settlement of larvae where amounts of SC were increased.

Most molluscan shells consist of a mineral portion that is more than 95% of the weight and an organic fraction that is less than 5% [51]. *C. gigas* shells contain more than 99% calcium carbonate and 0.5%, by weight, of some organic matrices [52]. These organic matrices in mollusks shells, which consist of polysaccharides and proteins, show great diversity in their molecular weights and amino acid compositions among species, and their secretion is fundamentally controlled by genes involved in shell formation [53]. The high settlement response of *C. gigas* larvae to shells of its own species and *C. nippona* suggests that larvae may recognize a settlement cue that may be abundant in conspecific shells and closely related species. In species with settlement inducing activities that were lower than *C. gigas* and *C. nippona*, the settlement cue may be present in the shells in smaller amounts. Thus, increasing the amount of shell given to the larvae can improve settlement inducing activity, as explained earlier in this discussion. Another explanation for the lower settlement response of *C. gigas* larvae to other species may be the difference in the matrix component of the shells. We are currently investigating the shell matrix compositions of the different shell species in order to check for possible relationships between shell matrix components and settlement inducing activities of different shell species.

The putative settlement cue in shells of conspecifics was stable even at 200°C and decreased in activity only when it was charred at 300°C (Fig. 4A). Knight-Jones [54] heated pieces of slates covered with bases of newly detached barnacles and observed that the activity of the settlement cue from *Balanus balanoides* shells remained unaffected until 200°C but this was gradually destroyed at about 250°C. The stability of some proteins from the shell matrix at high temperatures has also been demonstrated elsewhere [45], [55]. The settlement inducing activity of *C. gigas* SC also remained intact even after antibiotics treatment (Fig. 4B). Heat [56] and antibiotics [56]–[60] treatments have been used to kill constituent organisms in microbial biofilms, particularly bacterial constituents in the latter treatment. Consequently, these treatments resulted in the loss of biofilm and/or bacterial activity to induce larval settlement. The fact that *C. gigas* SC retained settlement inducing activity even after heat and antibiotic treatments suggests that the settlement cue was contained in *C. gigas* shell itself and distinct from the cue in bacterial biofilms reported in early studies [61].

Aqueous and organic solvent extracts of *C. gigas* shells did not significantly induce larval settlement at all concentrations tested, indicating that the chemical cue was not efficiently extracted by these solvents. By contrast, the HCl extract of *C. gigas* shells showed settlement inducing activity that was equivalent to the activity of the shell itself (Fig. 5). Therefore, HCl proved to be the most efficient solvent to use in extracting the settlement inducing compound in *C. gigas* shells. SC residues obtained after water and ethanol extractions exhibited lower activities as compared to SC (positive control) (Fig. 5), and this finding suggests the possibility of the presence of a settlement inducer compound(s) in both Aq-ex and EtOH-ex, although concentrations of Aq-ex and EtOH-ex tested in this investigation may not have been enough to significantly induce larval settlement. Moreover, during water extraction of SC, SC are crushed to a powdered state and therefore, this change in the physical property may also account for the decrease in settlement inducing activity of the residue obtained. Nevertheless, larval responses to Aq-ex, EtOH-ex and Et_2_O-ex warrant further investigation.

Acids are often used in the demineralization of shells to collect the protein matrix [45], [55], [62]. Results indicate that the acid-soluble matrix of *C. gigas* shells contained a settlement inducing compound for conspecific larvae. Moreover, a series of treatments (heat, enzymatic and chemical) experiments conducted on the acid-soluble settlement inducing compound suggested that the substance was a heat stable glycoprotein that was digested in pepsin and trypsin (Fig. 6), and deglycosylated with PNGase F and trifluoromethanesulfonic acid or TFMS (Fig. 7). Reduction in the activity of the settlement inducing compound after trypsin treatment also suggests the presence of arginine and/or lysine in the peptide because trypsin cleaves the carboxyl bond or arginine or lysine anywhere in a peptide [63]. Moreover, PNGase F cleaves between the GlcNAc and asparagine residues of *N*-linked oligosaccharides [64]. Gel filtration of the matrix compound of *C. gigas* shells resulted in the settlement cue being eluted between 45 and 150 kDa (Fig. 8). SDS-PAGE also confirmed the settlement inducing compound to have a major protein band with a molecular weight of 55 kDa (Fig. 9). The band was visualized after staining with Stains-all, which is used to dye phosphoproteins [65], [66]. Stains-all will also stain in blue those proteins that may have cation-binding potential [67].

The role of glycoproteins in the larval settlement has been described in the jellyfish *Cassiopea xamachana* [68], nudibranch *Aladaria proxima* [69] oyster *C. virginica* [33], [70], [71] and the sand dollar *Dendraster excentricus* [15]. In all these species, larvae responded to waterborne peptides of more than 1000 Da of MW [5]. In the barnacle *A. amphitrite*, the settlement inducing compound from adult conspecifics, referred to as Settlement Inducing Protein Complex or SIPC, is also a glycoprotein of high molecular mass consisting of three major subunits of 76, 88, and 98 kDa with lentil lectin (LCA)-binding sugar chains [20]. The glycoprotein in the organic matrix of *C. gigas* shells that acted as settlement cue was acid-soluble and insoluble in water, as demonstrated in the control groups of the treatment experiments (Figs. 6, 7) where the settlement cue was absorbed and remained intact in GF/C substrates even after washing with FSW. This insoluble settlement cue in *C. gigas* shells may account for the high recruitment of *C. gigas* on shells of dead conspecifics that was observed by Diederich [37] in the Oosterschelde estuary (Netherlands), and may be a chemical basis of larval settlement on conspecifics.

The insoluble nature of this cue suggests that the cue is recognized by *C. gigas* larva only after direct contact with the substrate. Hence, the swimming oyster larva may encounter the cue on a substrate (e.g. shells of conspecifics) through the mediation of other cues from the environment. Water soluble cues may play a role in the commencement of the settlement/searching behavior of the planktonic oyster larvae. Waterborne cues from bacteria [33], [72] and conspecifics [33], [73] have been reported to mediate the settlement behavior of oyster larvae. Tamburri et al. [33] reported that *C. virginica* larvae responded similarly to waterborne substances released both from adult oysters and from biofilms, that is, swimming in a manner indicative of settlement behavior. Coon et al. [74] demonstrated that *C. gigas* larvae exhibited settlement behavior that typically precedes cementation and metamorphosis when exposed to ammonia and suggested that ammonia may be a natural environmental cue. Fitt and Coon [75] reported that adult oysters produce enough NH_3_ needed to induce larval settlement behavior, and that concentrations of NH_3_ in the water near oyster shells in oyster beds is enough to trigger this behavior. Thus, Bonar et al. [76] suggested that if a larva enters an area of elevated NH_3_, as for example in a dense community assemblage such as an oyster bed, it begins the stereotyped searching behavior, and if the environment is acceptable, the additional cues the larva receives (tactile, physical, chemical, etc.) promote ultimate metamorphosis. Bao et al. [77] also suggested the involvement of two chemical cues, a waterborne and a surface bound cue, in the larval settlement of *M. galloprovincialis*. Further investigation may clarify possible synergistic influence of both waterborne and surface bound cues on larval settlement of *C. gigas*.

In conclusion, *C. gigas* larvae settled on shells of different species of mollusks but settled in higher numbers on shells of its conspecifics and *C. nippona*, suggesting that the cue may be more abundant or in a more available form to the larvae in shells of conspecifics and *C. nippona* than in other species. In *C. gigas* shells, the settlement cue was a heat stable 55 kDa glycoprotein component in the organic matrix that was soluble in acid. This is the first report of an insoluble glycoprotein in the organic matrix of *C. gigas* shells that acts as a chemical cue for larval settlement of conspecifics. The larval settlement cue that was extracted from adult *C. gigas* shells can also find application in the aquaculture industry of this economically important species. For example, this insoluble cue can be used to develop effective spat collectors of both wild and hatchery grown seeds by applying it on surfaces.
